# Editorial: A Toxic Aging Coin perspective to investigate the intersection of toxicology and aging

**DOI:** 10.3389/ftox.2024.1527706

**Published:** 2024-12-12

**Authors:** Rachel M. Wise, John P. Wise, Julie K Andersen, Michael Aschner

**Affiliations:** ^1^ Department of Pharmaceutical Sciences, University of New Mexico, Albuquerque, NM, United States; ^2^ Department of Pediatrics, Pediatrics Research Institute, University of Louisville, Louisville, KY, United States; ^3^ Buck Institute for Research on Aging, Novato, CA, United States; ^4^ Department of Molecular Pharmacology, Albert Einstein Collegeof Medicine, New York City, NY, United States

**Keywords:** toxic aging coin, environmental toxicology, aging, gerotoxicology, age differences

The intersection between toxicology and aging is a compelling yet often overlooked topic (Wise, 2022). Our society is imminently challenged with growing numbers of aging individuals across the globe as lifespans continue to lengthen and these geriatric populations continue increasing at an unprecedented rate (United Nations, 2019; Gu et al., 2021). Compounding this global aging challenge, understanding the contribution of environmental toxicants presents a critical two-sided dilemma that we have designated the “Toxic Aging Coin.” On the *heads* side we consider how age impacts effects of toxic chemicals, while on the *tails* side we consider how toxic chemicals exacerbate or accelerate aging outcomes (Wise, 2022). This volume compiles manuscripts on this timely topic exploring the dual nature of this intersection of aging and toxicology as a coin, one side representing the natural progression of life with changing toxic effects across life stages and the other considering the detrimental impacts of toxic exposure that can accelerate and complicate this journey.

Aging is a universal experience and the major risk factor for several neurodegenerative diseases, including Parkinson’s disease and Alzheimer’s disease. Aging is characterized by a gradual diminution of physiological processes that impairs the ability to reinforce internal homeostasis, and results in an increased vulnerability to disease, infections, trauma, and stress. This process is driven by genetic, environmental, and lifestyle factors; though the influence of genes on lifespan and aging Research Topic is estimated to be less than 10%, leaving the majority of influences to lifestyle and environmental factors (Ruby et al., 2018). The biological mechanisms underlying aging, such as telomere shortening, mitochondrial dysfunction, and cellular senescence, are well-documented. However, the role of toxic substances in modulating these mechanisms has been scantly characterized. To address the growing concerns that exposure to toxic substances can impact aging, interdisciplinary research is essential. Here, in several manuscripts/reviews we attempt to highlight the interaction between aging and toxicology.

Scieszka et al. approached this concept through the lens of exploring the role of wildfire smoke and air quality on aging processes. The authors of this review highlight key aging hallmarks, including telomere attrition and mitochondrial dysfunction, and how inhaled toxicants from wildfires may exacerbate these processes. Preliminary findings suggest wildfire smoke can accelerate neurological aging and impair learning by disrupting vascular integrity and promoting neuroinflammation. This review stresses the importance of studying the relationship between environmental exposures and aging mechanisms to inform future interventions, as well as the potential for using anti-inflammatory and NAD+ boosting compounds as intervention strategies.

An original research article by Tsai et al. reported that acute intoxication with organophosphate (OP) cholinesterase inhibitors can lead to a cholinergic crisis, resulting in severe symptoms including respiratory depression and seizures and that these health issues may evolve into further disease. Those who survive the acute effects often experience long-term behavioral deficits, but the mechanisms connecting acute OP exposure to chronic neurological issues remain unclear. The authors tested the hypothesis that acute OP intoxication induces cellular senescence in the brain using a rat model exposed to diisopropylfluorophosphate (DFP). While no signs of cellular senescence were found 1-month post-exposure, significant increases in the biomarker p16 were observed at three and 6 months, specifically in neurons within certain brain regions, suggesting cellular senescence as a potential mechanism for the long-term neurological deficits observed in survivors of OP intoxication.

A review from Ottinger et al. focuses on integrating the Toxic Aging Coin and One Health frameworks. The authors begin by introducing the One Health framework, which observes that the health of wildlife, ecosystems, and humans is interconnected, and emphasizing that wildlife preservation is essential for maintaining healthy ecosystems. They use examples including how sharp declines in avian populations over the last century due to human activities like industrialization, habitat loss, and climate change have led to increased stress and health issues for both humans and wildlife. The authors then present the concept of the “Toxic Aging Coin,” which suggests that environmental stressors not only accelerate aging but also affect older individuals’ ability to cope with pollutants. They then discuss how the concepts of the Toxic Aging Coin and One Health may be merged to explore important connections and propose strategies for improving health outcomes for wildlife and humans alike.

Finally, in a mini review, Ratner and Rutchik explore the relationship between copper exposure and neurological function, highlighting that both deficiencies and excesses can lead to adverse outcomes. They present a case study of a 53-year-old woman with early onset Lewy Body Dementia and Parkinsonism revealing chronic copper contamination in her drinking water, which may have contributed to her condition. This suggests that individuals at risk for α-synucleinopathies should avoid excessive copper exposure, similar to recommendations for those at risk of Alzheimer’s disease.

For the future, we recommend that together, toxicologists and gerontologists collaborate to investigate the influence of toxic substances on aging mechanisms as well as the role of age in the metabolism of toxic agents. It is imperative that future studies address the cumulative effects of toxicants throughout the lifespan to inform public health policies and regulations. In highlighting the intersection between toxicology and aging in these and similar studies, we also hope that this will encourage policymakers to better recognize the implications of toxic exposure on aging populations and to advocate for more comprehensive environmental health initiatives.
